# Synchronization of Non-linear Oscillators for Neurobiologically Inspired Control on a Bionic Parallel Waist of Legged Robot

**DOI:** 10.3389/fnbot.2019.00059

**Published:** 2019-08-02

**Authors:** Yaguang Zhu, Shuangjie Zhou, Dongxiao Gao, Qiong Liu

**Affiliations:** Key Laboratory of Road Construction Technology and Equipment of MOE, Chang'an University, Xi'an, China

**Keywords:** bionic parallel waist, locomotion control, synchronization, transition state, σ-Hopf oscillator

## Abstract

Synchronization of coupled non-linear oscillators inspired by a central pattern generator (CPG) can control the bionic robot and promote the coordination and diversity of locomotion. However, for a robot with a strong mutual coupled structure, such neurobiological control is still missing. In this contribution, we present a σ-Hopf harmonic oscillator with decoupled parameters to expand the solution space of the locomotion of the robot. Unlike the synchronization of original Hopf oscillators, which has been fully discussed, the asymmetric factor of σ-Hopf oscillator causes a deformation in oscillation waveform. Using the non-linear synchronization theory, we construct the transition state model of the synchronization process to analyze the asymmetrical distortion, period change and duty ratio inconsistency. Then a variable coupling strength is introduced to eliminate the waveform deformation and maintain the fast convergence rate. Finally, the approach is used for the locomotion control of a bionic parallel waist of legged robot, which is a highly coupled system. The effectiveness of the approach in both independent and synthesis behavior of four typical motion patterns are validated. The result proves the importance of controllability of the oscillation waveform and the instantaneous state of the synchronization, which benefits the transition and transformation of the locomotion and makes the coupling motion more flexible.

## Introduction

Legged robot technique has made remarkable progress in the past few decades, and produced a series of very famous achievements: StarlETH (Hutter et al., 2012), ANYmal (Hutter et al., 2016; Hwangbo et al., 2019), HyQ series (Semini et al., 2011, 2016), MIT Cheetah series (Seok et al., 2013; Wensing et al., 2017), Boston Dynamics Spotmini series^1^. Great contribution has been made in the aspects of leg structure (Ananthanarayanan et al., 2012), leg control (Barasuol et al., 2013), limb coordination (Gehring et al., 2016), compliance control (Eich et al., 2009), balance control (Raibert and Tello, 1986), adaptive control (Manoonpong et al., 2013), and so on (Schwendner et al., 2014; Gehring et al., 2016), which continuously improve the flexibility, stability, coordination and intelligence of robots in related fields.

No robot has been able to act as flexibly and as animal-like as cat or a dog until today. The control strategy is not the only reason for this, as there are many differences between the structure of natural creatures and robots. Inspired by biologists (Galis et al., 2014), along with demand for high-flexibility and high-speed robots, many scholars have carried out in-depth research on the bionic torso (Albiez et al., 2006). The bionic torso structure can be divided into two types: torso with active joint (Khoramshahi et al., 2013; Satoh and Fujimoto, 2018) and with passive joint (Takuma et al., 2010; Haynes et al., 2012). An additional active joint on a torso can effectively improve the motion range and dexterity of a robot. Most passive joints use elastic elements to collect energy during movement and to improve compliance. However, the function of a biological torso for load capacity, movement, coordination and other aspects is obviously much more than these. Therefore, this paper builds a bionic parallel waist to replace the original rigid torso for the advantages of strong carrying capacity and high control accuracy of the parallel structure. Actually, the parallel robot has been widely used in the industry fields (Dallej et al., 2006; Cong et al., 2011; He et al., 2015). Its structure (Villarreal-Cervantes et al., 2010), kinematics (Plitea et al., 2013), dynamics (Staicu, 2009), control (Liu et al., 2018b), and so on have all been studied before. Although the parallel structure has been directly applied to legged robot, showing better performance than serial structure (Kuehn et al., 2017), no bionical method is used on the parallel structure. This paper investigates the CPG-based network for neurobiologically inspired control of the bionic parallel waistf and discusses the related issues.

At present, CPG models have been applied in various robots (Donati et al., 2016; Santos et al., 2016; Liu et al., 2018a), and can be divided into two categories (Wu et al., 2009): neuron-based models and oscillatior-based models. Neuron-based models mainly include the Matsuoka neuron oscillator model (Matsuoka, 1985, 1987), the Kimura model (Kimura et al., 2001, 2002; Fukuoka et al., 2003), cellular neural network (CNNs) (Arena and Fortuna, 2000; Arena et al., 2001), and recurrent neural network (Rao and Kamat, 1995; Senda and Tanaka, 2000). The biological significance of the models is relatively clear. The oscillator-based models include the Kuramoto oscillator (Acebron et al., 2005), the Hopf harmonic oscillator (Righetti and Ijspeert, 2006; Righetti, 2008), the Van der Pol oscillator (Van der Pol and Van der Mark, 1928; Dutra et al., 2003), and more. These oscillator-based models can periodically generate non-linear oscillation signals and are widely used as CPG oscillation units since they contain a fewer number of parameters and have sophisticated background implementation theories. All of these above can be used for robot control and the networks with oscillators will not differ too much within the same robot, with respect to the architecture and coupling topology, which are related to the physical structure and control frame. In terms of waveforms that determine what trajectories will actually be performed by each joint during a cycle, the oscillator must be selected cautiously. In some robots with simple structure, waveform has a relatively small effect on the movement of single joint and whole body. Therefore, for the parallel platform in this paper, the spatial mechanism with six degrees of freedom is related to six coupling limbs. The movement of each limb directly affects the smoothness and stability of overall system, so we have to focus on the transition smoothness, transformation quickness, and velocity/acceleration impact of the oscillator.

Synchronization means an exact match of the scaled amplitude with a desired phase difference (Chung and Dorothy, 2010). Different phase lead or lag has already been performed in the oscillators with the same frequency to manipulate different locomotion of various robots (Ijspeert, 2008). There are two points that need to be figured out. One is the stability of the synchronization. The stable theory of the coupled oscillator system can help to determine the exact parameters, and they are the constraints that make the system converge at final moment (Buchli and Ijspeert, 2004; Kopell et al., 2006). This consideration has been fully discussed both in architectural symmetries and diffusion-like couplings (Buono and Golubitsky, 2001; Ashwin, 2003; Ramezani et al., 2017). Another point is that the transition process needs to be clear and controllable, since it concerns the trajectory of every movement of the limbs and joints. An unreasonable transition between two perfect stable states will also lead to an unexpected locomotion. In fact, in the premise of synchronization stability, the transition process in both joint space and Cartesian space should be taken into serious consideration. In this paper, the transition state of coupled non-linear oscillators is analyzed in detail to guarantee the rational locomotion in every single moment.

In our previous work, we have researched on legged robot about trajectory planning (Zhu and Guo, 2016; Zhu et al., 2018a), deviation correction (Zhu et al., 2016, 2017), force control (Zhu et al., 2015; Zhu and Jin, 2016), energy optimization (Jin et al., 2013; Zhu et al., 2014), and locomotion diversity (Zhu et al., 2018b), aiming to achieve excellent coordination and flexibility of legged robot. The bionic parallel waist is supposed to be a good choice for the issue from both a biological and a robotic point of view. At present, the majority of parallel robots utilize the model control theory (Jin et al., 2014). Each angle of joint with the strong coupling and structure constraints needs to be calculated, since all joint actuators are involved in the locomotion of parallel robots at the same time. All trajectories (position, velocity, acceleration, and torque) have to be designed for accurate control and high speed. Although these methods can be used in the bionic robot, we are more inclined to the bionic control method, for it is hard to design every possible movement in the solution space in advance. Thus, synchronization of σ-Hopf oscillators for the bionic parallel waist of a legged robot is proposed to realize neurobiologically inspired control. The paper is organized as follows. Materials and Method introduces the bionic parallel robot system and the method. In Analysis of Synchronization, synchronization of σ-Hopf oscillators is analyzed. Neurobiologically Inspired Control and Results illustrates CPG-Based locomotion control and results. The performances and findings are discussed and concluded in Simulation.

## Materials and Methods

For the coordinated locomotion of a legged robot, a bionic parallel waist is developed for the method proposed in this paper. We will introduce the system of the parallel waist and synchronization method of coupled σ-Hopf oscillators in the following part: sections Platform and System, σ-Hopf Harmonic Oscillator, Network, and Synchronization of σ-Hopf Oscillators.

### Platform and System

**Figure 1:**
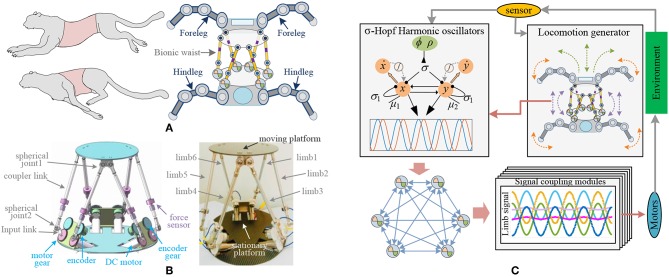
Architecture of bio-inspired legged robot with parallel waist. **(A)** Mechanical structure of bio-inspired robot. **(B)** Mechanical structure of bionic waist. **(C)** Framework of robot system, including locomotion generator of the robot, σ-Hopf oscillators, synchronous network, and coupling signal modules.

In mammals, the waist (both bone and muscle) plays an extraordinary role in movement (Grondin and Potvin, 2009), and researchers found that robots with waists performed better than those with a rigid body (Coros et al., 2011). The parallel platform in this paper is used as the waist of the four-legged robot to obtain better coordination ability. Its structure is shown in Figure 1A. The moving platform at the waist connects the front legs, while the rear legs are linked to the stationary platform. The whole parallel platform is supposed to be the robot torso and can achieve shift, rotation, and synthesis movements. Six identical limbs connecting the moving platform to the stationary platform are the actuating mechanism of the waist. There are two spherical joints between coupler links, and one rotation joint for the connection of an input rod and the motor. So, the moving platform can move with 6 spatial degrees of freedom effectively under the actuation of 6 brushless DC motors. The magnet encoders are mounted next to the input rods and are connected by gears. They are shown in Figure 1B. Force sensors are mounted on the long linkages and used for the payload calculation. All information obtained through encoders and force sensors are sent to the controller for locomotion generation.

Most existing legged robots without waist structures adjust the action of the rigid torso by controlling the leg movement (Cao and Poulakakis, 2016; Zhang et al., 2016). Some others enhance the movement ability by active or passive joints. But the presented parallel waist has far more flexibility and larger load capacity, which can easily assist the robot to achieve pitching, stretching, and torsion of the torso. Therefore, the robot equipped with a bionic waist needs to carry out overall behavior planning including torso and legs, so as to generate motion rhythm and control parameters for the waist. With the bionic control method, these parameters will be used to adjust the CPG oscillator and synchronous network, and then generate the control signal for the motor driver, which is shown in Figure 1C.

### σ-Hopf Harmonic Oscillator

The important role of CPG in bionic control is self-evident and the details have been investigated in the field of biology (Macintosh et al., 1993; Jorgensen et al., 2003), neuroscience (Grillner et al., 2007; Jean-Xavier and Perreault, 2018), and robotics (Wang et al., 2012). Especially in the application of multi-degree-of-freedom or multi-joint robots, it is particularly prominent due to the coupling and synchronization ability of CPG signals, which can form a signal transmission network similar to natural creature. However, no matter how complex the network is or what change post-processing makes, the controllability of the original signal generated by CPG affects the control performance of robots directly. Then we must control CPG signal at every single moment. That is to say, if CPG is regarded as a simple control wave, we should not only pay attention to its frequency and amplitude, but also control its offset, the speed of ascending, and descending (shape of the signal). They are not problems in the model control (Jin et al., 2014), since all of the required control variables can be “designed out.” CPG waveform in the bionic control is subject to the control of oscillation and coupling property, and cannot be changed easily (Zhu et al., 2018b). In the present paper, the σ-Hopf oscillator is proposed [σ is one special parameter to distinguish it from the orignal Hopf oscillator (Drazin, 2008)] for two reasons. One is the Stability. The symmetric limit circle prevents the classical problem of instability due to switching between two stable systems and the stability of coupled Hopf oscillators has also been previously discussed (Pham and Slotine, 2005; Kato and Kamimura, 2008). The other reason is the consideration for excellent locomotion diversity and smoothness by changing amplitude, frequency, phase, and waveform, which has been fully investigated in our published paper (Zhu et al., 2018b). Its features and characteristics will be introduced in the following. The form of synchronous coupling of σ-Hopf is

(1) $$\begin{array}{r} \overset{∙}{\upsilon }=\left[\begin{array}{c} ẋ\\ ẏ \end{array}\right]=\left[\begin{array}{c} \sigma_{1}\left(x-a\right)-\sigma \left(y-b\right)\\ \sigma_{1}\left(y-b\right)+\sigma \left(x-a\right) \end{array}\right]+\text{g}\left(t\right)+\text{u}\left(t\right) \end{array}$$

where,

(2) $$\{\begin{array}{c} \sigma =\pi /(\rho \cdot (e^{-\lambda y}+1)\cdot \varphi )\;+\pi /((1-\rho )\cdot (e^{\lambda y}+1)\cdot \varphi )\\ \sigma_{1}=-\alpha ({\left(x-a\right)}^{2}+{\left(y-b\right)}^{2}-\mu ) \end{array}$$

Equivalently,

(3) $$\begin{array}{r} \overset{∙}{\upsilon }=\text{f}\left(\upsilon ,\alpha ,\mu ,\sigma \left(\rho ,\lambda ,t\right)\right)+\text{g}\left(t\right)+\text{u}\left(t\right) \end{array}$$

where *x* and *y* are the state variables; *a, b* is the center of the limit cycle; μ is amplitude of the oscillations. The bifurcation parameter α can switch from −1 to 1 such that this would change the stable limit cycle dynamics to the dynamics with a globally stable equilibrium point (Strogatz, 2015). In the **σ**(ρ, λ, *t*), λ is strength and φ is the period factor; 0 < ρ <1 denotes the duty factor and determines the transformation speed between the ascending and descending phases. In (2), the parameters φ and ρ are uncorrelated. That means that the movement period will not be influenced by a change in the duty factor. **g**(*t*) is coupling input, for a single oscillator **g**(*t*) = 0. **u**(t) = –sign(*y*)*u* is the external input to control the oscillation signal by the external sensing information (i.e., contact detection in the legged robot; Righetti, 2008; Aoi et al., 2013). The coverage coefficient and frequency were always coupled together and cannot be controlled independently. Therefore, we decoupled it and induced the duty factor ρ so that the frequency and shape of the waveform can be controlled independently (Zhu et al., 2018b). The presence of **σ**(t) here is different from the original Hopf oscillator and plays an important role in changing the oscillation waveform. We will later see that they can be useful for different patterns of locomotion. In the next section, we present how to construct a stable synchronization of σ-Hopf oscillators for the purpose of diverse locomotion pattern modulation.

### Network

**Figure 2:**
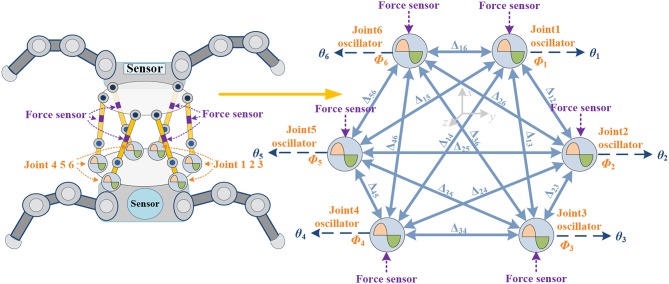
Two-way ring architecture network. Every limb composes of 2 links, 2 spherical hinges, 1 actuator, and 1 force sensor. Φ_*i*_ is denoted as oscillation phase of *i*th joint, θ_*i*_ is the control signal of *i*th joint. Geometric structure is shown in Appendix (Supplementary Material).

Synchronization permits different actuators to oscillate with a prescribed phase lead or lag to regulate different movement patterns. On the basis of the joint number, structure connection and behavioral characteristics of different robots [legged robot (Kalouche et al., 2015; Bjelonic et al., 2016; Owaki and Ishiguro, 2017), snake robot (Wang et al., 2016), swimming robot (Stefanini et al., 2006), and flying robot (Corke et al., 2003; Ramezani et al., 2017)], the behavior control networks are also different. The type of oscillator (Wang et al., 2018) and phase difference (Aoi et al., 2011) formation method are also important factors. The specific network architecture is shown in Figure 2. The parallel waist platform adopts the same control frame as the leg of the robot, and each limb is driven by an oscillator. The full coupled two-way ring architecture is constructed, since it has better transition performance than one-way ring architecture (Chung and Dorothy, 2010). For the coupled σ-Hopf oscillators in this paper, (3) can be rewritten with a diffusive coupling with the phase-rotated neighbor

(4) $$\begin{array}{r} \overset{∙}{\upsilon_{i}}=\text{f}\left(\upsilon_{i},\mu_{i},\sigma_{i}\left(\rho_{i},\lambda ,t\right)\right)\\ \;-k\left(t\right)\overset{n_{i}}{\underset{j\in {𝔑}_{i}}{\sum }}\left(\upsilon_{i}-\frac{\mu_{i}}{\mu_{j}}\text{R}\left(\Delta \Phi_{ij}\right)\upsilon_{j}\right)+\text{u}\left(t\right) \end{array}$$

(5) $$\begin{array}{r} \text{R}\left(\Delta \Phi \right)=\left[\cos\left(\Delta \Phi \right)-\sin\left(\Delta \Phi \right);\sin\left(\Delta \Phi \right)\cos\left(\Delta \Phi \right)\right] \end{array}$$

in which, the positive scalar *k*(t) denotes the coupling gain and can be time-varying for different locomotion. **R**(ΔΦ)SO(2) is a 2-D rotational transformation of the phase difference ΔΦ_*ij*_ between the *i*th and *j*th oscillators. The desired phase offset ΔΦ_*ij*_ guides υ_*i*_ to synchronize with υ_*j*_. N_*i*_ denotes the set that contains only the local neighbors of the *i*th σ-Hopf oscillator, and *n*_*i*_ is the number of the neighbors. Both N_*i*_ and *n*_*i*_depend on the coupled σ-Hopf oscillators and network architecture. Since the coordination motion pattern of the bionic waist is determined by the relative phase between the oscillators, the phase offset matrix ΔΦ_*ij*_ is the key in the network. Generally, ΔΦ_*ij*_ = Φ_*i*_ – Φ_*j*_ (*i, j* = 1,…,6, 0 ≤ ΔΦ_*ij*_ ≤ 2π), ΔΦ_*ij*_ = –ΔΦ_*ji*_, ΔΦ_*ij*_ = ΔΦ_*ik*_ + ΔΦ_*kj*_ and ΔΦ_*ii*_ = 0 (*i, j* = 1,…,6). So, the locomotion of the waist is determined by [ΔΦ_12_, ΔΦ_23_, ΔΦ_34_, ΔΦ_45_, ΔΦ_56_, ΔΦ_61_], which can be written as [Δ_12_, Δ_23_, Δ_34_, Δ_45_, Δ_56_, Δ_61_] for simplification. The full coupled network with ΔΦ is shown in Figure 2.

Theoretically, a 6RSS parallel platform can realize the independent and coupled locomotion of three translational and three rotational motions in the Cartesian coordinate system (Ahmet and Koksal, 2014) In this paper, four main torso movements of the mammals (Rossignol, 2004) are discussed: Stretching and flexing along Z axial (motion pattern A), lateral movement along X axial (motion pattern B), pitch around Y axial (motion pattern C), and torsion around Z axial (motion pattern D). According to the symmetry of motion and the established coordinate system shown in Appendix (Supplementary Material), the oscillators always operate synchronously. From a lateral perspective, all mentioned actions are symmetrical along the X axial except rotation movement around the Z axial. The joint 1–6, 2–5, and 3–4 have the opposite turning direction to guarantee the symmetric loading, so Δ_16_ = Δ_25_ = Δ_34_ = 180° (for the rotation around the Z axial, Δ_16_ = Δ_25_ = Δ_34_ = 0°). The oscillator amplitude is also symmetric along the X axial. Vertically, in the motion pattern A, oscillators on the same side along the X axial keep same pace, then Δ_12_ = Δ_23_ = 0°and Δ_65_ = Δ_54_ = 0°; in motion pattern B, there are Δ_12_ = Δ_65_ = 0° and Δ_23_ = Δ_54_ = 180°according to the asymmetric structure along the Y axial; in motion pattern C, there are Δ_16_ = Δ_25_ = Δ_34_ = 180°, Δ_12_ = Δ_65_ = 180° and Δ_23_ = Δ_54_ = 0°, since the actuator 1 and 6 are located in the positive half of X axial and joint 2 (or 3) and 5 (or 4) are located in the negative half; in motion pattern D, all actuators have the same rotational direction, so Δ_16_ = Δ_25_ = Δ_34_ = 0°, Δ_12_ = Δ_65_ = 180°, Δ_23_ = Δ_54_ = −180° and Δ_13_ = Δ_64_ = 0°. Other independent or coupled locomotions can be obtained by similar analysis, even if they never occur in vertebrates. Additionally, the range of movements are associated with the amplitude control signals. We will illustrate these relationships in Neurobiologically Inspired Control and Results.

### Synchronization of σ-Hopf Oscillators

The neurobiological approach to an engineered bionic waist is to produce the analytical model of oscillators that matches the real need of bioinspired legged robot. It will benefit the coordinated locomotion between the waist and leg joints, but the limit-cycle dynamics, synchronization of the coupled oscillator and feedback signals integration have to be taken into consideration (Ijspeert, 2008; Seo et al., 2010). Thus, we have proposed (1) with period parameter ϕ, amplitude μ, coupled input ***g***(t), and feedback input ***u***(t). This is the base for the bionic waist to perform agile maneuvering in decoupled, symmetric or unsymmetric locomotion in Cartesian space. For the synchronization of a network, we rewrite the coupled Hopf oscillators with the related concern:

(6) $$\begin{array}{r} \overset{∙}{\upsilon_{i}}=\text{f}\left(\upsilon_{i},\mu_{i},\sigma_{i}\left(\rho_{i}\right)\right)-k\left(t\right)\overset{n_{i}}{\underset{j\in {𝔑}_{i}}{\sum }}\left(\upsilon_{i}-\frac{\mu_{i}}{\mu_{j}}\text{R}\left(\Delta \Phi_{ij}\right)\upsilon_{j}\right). \end{array}$$

If the external sensing input *u*(t) is taken as some initial state of the oscillator, its value will not affect the stability. If σ equals to a constant or ρ = 0.5, ascending and descending will equal to each other, it will be the standard Hopf oscillator, and its stability with a diffusive coupling has been proved (Pham and Slotine, 2005). When ρ≠0.5, the σ-Hopf oscillation waveform is asymmetrical. Although the total time in one period is still unchanged, it presents different frequencies (π/(ρ·(*e*^−λ*y*^+1)·φ) for ascending and π/((1-ρ)·(*e*^λ*y*^+1)·ϕ) for descending), and leads to asymmetrical oscillation signals. Thus, to demonstrate the synchronization of the modified oscillators, $\left\{\overset{∙}{\bar{\upsilon }}\right\}$ is denoted for the symmetric oscillators (with ρ = 0.5). According the stable theory of phase synchronization, we construct (6) into

(7) $$\begin{array}{r} \left\{\overset{∙}{\bar{\upsilon }}\right\}=\text{f}\left(\left\{\bar{\upsilon }\right\},\mu ,\sigma^{\rho =0.5}\right)-k\left(t\right)\text{G}\left\{\bar{\upsilon }\right\} \end{array}$$

where ρ ∈ ℝ^n^, and different ρ makes synchronization of oscillators with different wave shape possible. Specially, if ρ = 0.5, the shape of waveform will equal to the original waveform. The matrix **G** is a Laplacian matrix with phase shifts **R**(ΔΦ_*ij*_). Considering instantaneous deformation, $\left\{{\bar{\upsilon }}_{0}\right\}$ is denoted for the original oscillators signal without synchronization, $\left\{\bar{\upsilon }\right\}$ is for the original oscillators signal with synchronization, and$\left\{\Delta {\bar{\upsilon }}_{s}\right\}$ is the change between them. So

(8) $$\begin{array}{r} \bar{\upsilon }={\bar{\upsilon }}_{0}+\Delta {\bar{\upsilon }}_{s}\left(\text{G}\left({\bar{\upsilon }}_{0}\right)\right) \end{array}$$

For simplicity, $\Delta {\bar{\upsilon }}_{s}\left(\text{G}\left({\bar{\upsilon }}_{0}\right)\right)$is written as$\Delta {\bar{\upsilon }}_{s}$,

(9) $$\begin{array}{l} \left\{\overset{∙}{\bar{\upsilon }}\right\}=\text{f}\left(\left\{\bar{\upsilon }\right\},\mu ,\sigma^{\rho =0.5}\right)-k\left(t\right)\text{G}\left\{\bar{\upsilon }\right\}=\text{f}\left(\left\{{\bar{\upsilon }}_{0}\right\},\mu ,\sigma^{\rho =0.5}\right)\\ \;+\text{f}\left(\left\{\Delta {\bar{\upsilon }}_{s}\right\},\mu ,\sigma^{\rho =0.5}\right)-k\left(t\right)\text{G}\left\{{\bar{\upsilon }}_{0}\right\}-k\left(t\right)\text{G}\left\{\Delta {\bar{\upsilon }}_{s}\right\}\\ \;=\text{f}\left(\left\{{\bar{\upsilon }}_{0}\right\},\mu ,\sigma^{\rho =0.5}\right)+G\left\{\text{G}\left({\bar{\upsilon }}_{0}\right),k\left(t\right)\right\} \end{array}$$

(10) $$\begin{array}{r} G\left\{\text{G}\left({\bar{\upsilon }}_{0}\right),k\left(t\right)\right\}=\text{f}\left(\left\{\Delta {\bar{\upsilon }}_{s}\right\},\mu ,\sigma^{\rho =0.5}\right)-k\left(t\right)\text{G}\left\{{\bar{\upsilon }}_{0}\right\}\\ \;-k\left(t\right)\text{G}\left\{\Delta {\bar{\upsilon }}_{s}\right\}. \end{array}$$

When finally it gets stable, {**υ**} → {**υ**_0_}. Then,

$$\begin{array}{r} \left\{\overset{∙}{\bar{\upsilon }}\right\}=\text{f}\left(\left\{{\bar{\upsilon }}_{0}\right\},\mu ,\sigma^{\rho =0.5}\right)+G\left\{{\bar{\upsilon }}_{0},k\left(t\right)\right\} \end{array}$$

(11) $$\{\begin{array}{c} \{\Delta {\bar{\upsilon }}_{s}\}=0\\ f(\{\text{Δ}{\bar{\upsilon }}_{0}\},\mu ,\sigma^{\rho =0.5})=0\\ G\{G({\bar{\upsilon }}_{0}),k(t)\}=0 \end{array}$$

So

(12) $$G\{G({\bar{\upsilon }}_{0}),k(t)\}\{{}_{=0,steadystate}^{\neq 0,transitionstate}$$

It shows that the signals of oscillators with synchronization in the transition state are different from that of original oscillators, but they will turn back to their original form after the convergence.

The modified signals of oscillators (with ρ≠0.5) without synchronization is denoted as $\left\{{\hat{\upsilon }}_{0}\right\}$ and {Δ**υ**_0_}is the deviation between $\left\{{\bar{\upsilon }}_{0}\right\}$ and $\left\{{\hat{\upsilon }}_{0}\right\}$. According to (8), for modified signal with synchronization

(13) $$\begin{array}{r} {\hat{\upsilon }}_{0}={\bar{\upsilon }}_{0}+\Delta {\hat{\upsilon }}_{0} \end{array}$$

(14) $$\begin{array}{r} \hat{\upsilon }={\hat{\upsilon }}_{0}+\Delta {\hat{\upsilon }}_{s}\left({\hat{\upsilon }}_{0}\right)={\hat{\upsilon }}_{0}+\Delta {\hat{\upsilon }}_{s}\left({\bar{\upsilon }}_{0},\Delta {\hat{\upsilon }}_{0}\right). \end{array}$$

$\Delta {\hat{\upsilon }}_{s}\left({\bar{\upsilon }}_{0},\Delta {\hat{\upsilon }}_{0}\right)$ is written as $\Delta {\hat{\upsilon }}_{s}$. For $\left\{\overset{∙}{\hat{\upsilon }}\right\}$, we have

(15) $$\begin{array}{r} \left\{\overset{∙}{\hat{\upsilon }}\right\}=\left\{{\overset{∙}{\bar{\upsilon }}}_{0}\right\}+\left\{\Delta {\overset{∙}{\hat{\upsilon }}}_{0}\right\}+\left\{\Delta {\overset{∙}{\hat{\upsilon }}}_{s}\right\}=\left\{{\overset{∙}{\hat{\upsilon }}}_{0}\right\}+\left\{\Delta {\overset{∙}{\hat{\upsilon }}}_{s}\right\} \end{array}$$

(16) $$\begin{array}{r} \left\{{\overset{∙}{\bar{\upsilon }}}_{0}\right\}=\text{f}\left(\left\{{\bar{\upsilon }}_{0}\right\},\mu ,\sigma^{\rho =0.5}\right)-k\left(t\right)\text{G}\left\{{\bar{\upsilon }}_{0}\right\} \end{array}$$

(17) $$\begin{array}{r} \left\{\Delta {\overset{∙}{\hat{\upsilon }}}_{0}\right\}=\text{f}\left(\left\{\Delta {\hat{\upsilon }}_{0}\right\},\mu ,\sigma^{\rho =0.5}\right)-k\left(t\right)\text{G}\left\{\Delta {\hat{\upsilon }}_{0}\right\} \end{array}$$

(18) $$\begin{array}{r} \left\{\Delta {\overset{∙}{\hat{\upsilon }}}_{s}\right\}=\text{f}\left(\left\{\Delta {\hat{\upsilon }}_{s}\right\},\mu ,\sigma^{\rho \neq 0.5}\right)-k\left(t\right)\text{G}\left\{\Delta {\hat{\upsilon }}_{s}\right\} \end{array}$$

(19) $$\begin{array}{r} \left\{{\overset{∙}{\hat{\upsilon }}}_{0}\right\}=\text{f}\left(\left\{{\bar{\upsilon }}_{0}\right\},\mu ,\sigma^{\rho =0.5}\right)-k\left(t\right)\text{G}\left\{{\bar{\upsilon }}_{0}\right\}\\ \;+\text{f}\left(\left\{\Delta {\hat{\upsilon }}_{0}\right\},\mu ,\sigma^{\rho =0.5}\right)-k\left(t\right)\text{G}\left\{\Delta {\hat{\upsilon }}_{0}\right\} \end{array}$$

(20) $$\begin{array}{r} \left\{\overset{∙}{\hat{\upsilon }}\right\}=\text{f}\left(\left\{{\bar{\upsilon }}_{0}\right\},\mu ,\sigma^{\rho =0.5}\right)+\text{f}\left(\left\{\Delta {\hat{\upsilon }}_{0}\right\},\mu ,\sigma^{\rho =0.5}\right)\\ \;+G'\left\{{\bar{\upsilon }}_{0},\Delta {\hat{\upsilon }}_{0},k\left(t\right)\right\} \end{array}$$

(21) $$\begin{array}{r} G'\left\{{\bar{\upsilon }}_{0},\Delta {\hat{\upsilon }}_{0},k\left(t\right)\right\}=\text{f}\left(\left\{\Delta {\hat{\upsilon }}_{s}\right\},\mu ,\sigma^{\rho \neq 0.5}\right)-k\left(t\right)\text{G}\left\{{\bar{\upsilon }}_{0}\right\}\\ \;-k\left(t\right)\text{G}\left\{\Delta {\hat{\upsilon }}_{0}\right\}-k\left(t\right)\text{G}\left\{\Delta {\hat{\upsilon }}_{s}\right\}. \end{array}$$

Remark:

1. If $\Delta {\hat{\upsilon }}_{0}$ ≠0 and *k*(t) ≠ 0, the process will be signal synchronization of modified Hopf oscillators. For any fixed parameters, the deformation $\Delta {\hat{\upsilon }}_{0}$ of ${\hat{\upsilon }}_{0}$ and ${\bar{\upsilon }}_{0}$ will be a constant, so $\text{G}\left\{\Delta {\hat{\upsilon }}_{0}\right\}$ ≠ 0. The related $\Delta {\hat{\upsilon }}_{s}\left({\bar{\upsilon }}_{0},\Delta {\hat{\upsilon }}_{0}\right)$ will be determined by $k\left(t\right)\text{G}\left\{{\bar{\upsilon }}_{0}\right\}+k\left(t\right)\text{G}\left\{\Delta {\hat{\upsilon }}_{0}\right\}$ and the amplitude is also fixed, so G' is not equal to 0. Under the influence of G', modified oscillators signal with synchronization will be different from original signal (ρ = 0.5), and the deformation depends on the specific parameters ρ and *k*. Also, for any parameter *k*(t) satisfying the stability condition (Pham and Slotine, 2005), ${\hat{\upsilon }}_{0}$ will have a corresponding stable state.
2. If $\Delta {\hat{\upsilon }}_{0}$ = 0, *k*(t) ≠ 0, thus ${\hat{\upsilon }}_{0}={\bar{\upsilon }}_{0}$ and $\left\{\;\overset{∙}{\hat{\upsilon }}\right\}=\left\{{\overset{∙}{\bar{\upsilon }}}_{0}\right\}$, it means they are original oscillators signal with synchronization, $k\left(t\right)\text{G}\left\{{\bar{\upsilon }}_{0}\right\}+k\left(t\right)\text{G}\left\{\Delta {\hat{\upsilon }}_{0}\right\}$ will 0, then $\Delta {\hat{\upsilon }}_{s}\left({\bar{\upsilon }}_{0},\Delta {\hat{\upsilon }}_{0}\right)=0$, G' is equal to G, and suitable *k*(*t*) will make it global stable.
3. Usually, in the synchronization, the convergence strength *k*(t) ≠0. But if *k*(*t*) is approaching 0 gradually and the time is long enough for stabilization with a certain value of it, things will be different. We assume that *k*_*n*__+1_ < *k*_*n*_ for ∀ *n*, Then, every part of G' in (21) can be constructed in stable amplitude form^*n*^

(22) $$\begin{array}{r} H_{G}^{n}=H_{\text{f}}^{n}-H_{G}^{n}-H_{0}^{n}-H_{s}^{n}, \end{array}$$

(23) $$\begin{array}{r} H_{\text{f}}^{n}\propto \left(H_{0}^{n},H_{s}^{n}\right)\;and\;H_{G}^{n}=0. \end{array}$$

Then,

(24) $$\begin{array}{r} k\to 0\;\Rightarrow H_{0}^{},H_{s}^{}\to 0\Rightarrow H_{\text{f}}^{}\to 0\Rightarrow H_{G}^{n}. \end{array}$$

So every change of *k* will have a new $H_{G}^{n}$ and it will never equal to 0 before stable. It seems very hard to let $H_{G}^{n}\to 0$, since it is hard to know how long it needs to get stable. But if the small deformation of waveform is acceptable, large convergence rate will be achieved. Thus, there is a trade off between convergence rate and final deformation, and the details will be discussed as follows.

## Analysis of Synchronization

Through the transition state analysis of the synchronization process, *k*(t) is the only parameter which can be adjusted, which determines the convergence rate and the steady state of the oscillator synchronization. For the original symmetric hopf oscillator, *k*(t) can be large to meet the requirement of rapid convergence, while for σ-hopf oscillator, whose waveform can be asymmetric, the process will be different and specific analysis is required.

**Figure 3:**
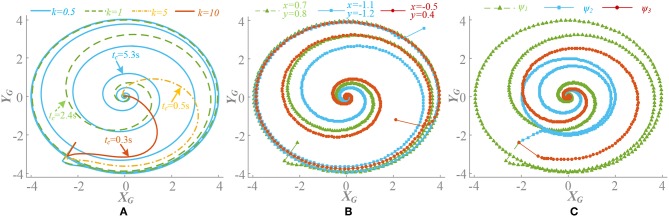
Convergence process of $G\left\{G\left({\bar{\upsilon }}_{0}\right),k\left(t\right)\right\}$with influence of different parameter (ρ = 0.5). Signal of limb 1 is selected. **(A)** Different value of *k*(t), phase factors ψ_*i*_ = Φ_*i*_/2π, ψ = [0, 0.5, 0.5, 0, 0, 0.5], initial point is (0.7, 0.8). Different *k*(t) leads a different convergence rate. **(B)** Different initial points (*x*_0_, *y*_0_), *k*(t) = 1, ψ = [0, 0.5, 0.5, 0, 0, 0.5]. Initial point has little influence on convergence rate, but is important to synchronization process. **(C)** Different phase factors ψ_1_ = [0, 0.5, 0.5, 0, 0, 0.5], ψ_2_ = [0, 0.5, 0, 0.5, 0, 0.5], ψ_3_ = [0.2, 0.4, 0.6, 0.1, 0.9, 0.7]. *k*(t) = 1, initial point is (0.7, 0.8). A different phase factor means a different synchronization process.

**Figure 4:**
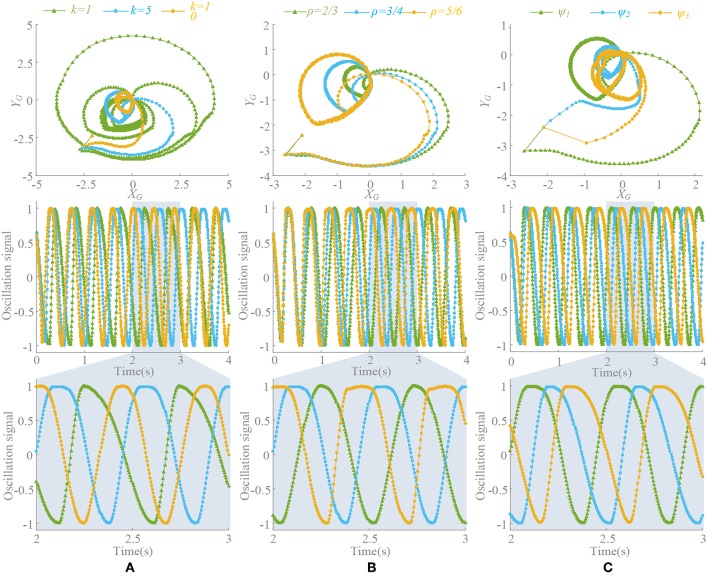
Influence of different parameters with ρ≠0.5. Signal of limb 1 is selected. **(A)** Different value of *k*(t), phase factors ψ_*i*_ = Φ_*i*_/2π, ψ = [0, 0.5, 0.5, 0, 0, 0.5], ρ = 3/4 and initial point (0.7, 0.8). Different *k*(t) leads a different limit circle scale of $G\left\{{\bar{\upsilon }}_{0}\right\}$ and the deformation is directly related to $k\left(t\right)G\left\{{\bar{\upsilon }}_{0}\right\}$. **(B)** Different **ρ** = [2/3, 3/4, 5/6], initial point (0.7, 0.8), and *k*(t) = 5. Large ρ leads to large deformation. **(C)** Different phase factors ψ_1_ = [0, 0.5, 0.5, 0, 0, 0.5], ψ_2_ = [0, 0.5, 0, 0.5, 0, 0.5], ψ_3_ = [0.2, 0.4, 0.6, 0.1, 0.9, 0.7], *k*(t) = 5, initial point (0.7, 0.8), and ρ = 3/4. Different motion pattern has different limit circle of $G\left\{{\bar{\upsilon }}_{0}\right\}$.

**Figure 5:**
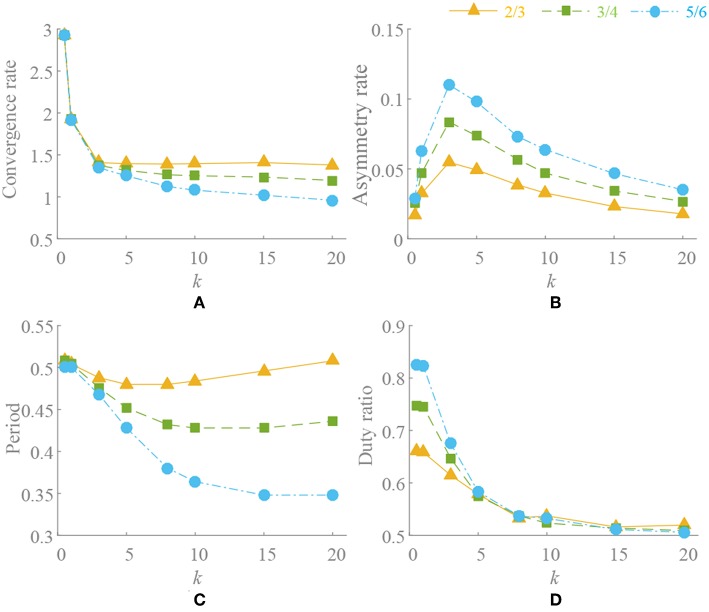
Convergence and deformation of waveform. Oscillation period is set to *T* = 0.5s. ***k*** = [0.5, 1, 3, 5, 8, 10, 15, 20], **ρ** = [2/3, 3/4, 5/6]. **(A)** Convergence rate. **(B)** Asymmetry rate. **(C)** Period change. **(D)** Duty ratio.

1. When ρ = 0.5, there is $\Delta {\hat{\upsilon }}_{0}$ = 0. *k*(t) is convergence strength and $G\left\{\text{G}\left({\bar{\upsilon }}_{0}\right),k\left(t\right)\right\}$ converges to zero. The different value of *k*(t), initial points and phase factors will cause different convergence rates. The convergence process of $G\left\{\text{G}\left({\bar{\upsilon }}_{0}\right),k\left(t\right)\right\}$is shown in Figure 3. Generally speaking, on the premise of global stable, the larger *k*(t) leads to faster convergence rate, as well as motion pattern transformation. If the initial point is designed close to the limit cycle with the concern of synchronization, it also contributes to the fast convergence rate. The phase factors reflect the synchronization relation of the oscillators and determine the phase difference at steady state.
2. When ρ ≠ 0.5, there is $\Delta {\hat{\upsilon }}_{0}$ ≠ 0. Through the separate adjustment of *k*, ρ and phase factors, $\text{G}\left\{{\bar{\upsilon }}_{0}\right\}$ whose trend is consistent with $G'\left\{\text{G}\left({\bar{\upsilon }}_{0}\right),k\left(t\right)\right\}$, is analyzed. The different process of approaching to a steady state is shown in Figure 4. As in the previous analysis, $k\left(t\right)\text{G}\left\{{\bar{\upsilon }}_{0}\right\}$ does not equal 0 in a steady state and it also stabilizes to a non-circular asymmetrical limit cycle, which brings about an asymmetrical oscillation waveform as analyzed by (21). For a certain motion pattern with fixed coupling parameter, only the effects of the *k* and ρ on the oscillator need to be analyzed. For example, in motion pattern B, the oscillators on the left and right side along the X axial are symmetrical, so taking the oscillator 1 as a representative. The convergence rate, asymmetry rate (the difference between the positive and negative areas, 0 means no difference), period change and duty ratio (the ratio of ascending part in one period cycle, 0.5 represents the symmetry waveform) of the steady-state waveforms with different *k* and ρ are shown in Figure 5. The convergence rate is mainly determined by *k*. The convergence is very slow in *k* <1 with almost no distortion, fast in *k* = 1–5 with a little distortion and extremely fast in *k* > 5 with large distortion and period change. After *k* = 10, convergence rate should have been <1 s (steady state is judged by checking the change of peak values in adjacent period). Besides, there are two findings: (1) large ρ value brings about large distortion, as shown in Figures 4B, 5B. (2) In the raise of *k* value, the waveform tends to be symmetrical (asymmetry rate tends to 0 and the duty ratio tends to 0.5) with period shrinking. That means the entire waveform is changed to be “small” and “smooth,” which is much different from expected, as shown in Figures 4A, 5C,D.

Since the value of *k*(t) determines the convergence rate and the magnitude of the distortion, a function *k*_e_(t) is proposed:

(25) $$\begin{array}{r} k_{e}\left(t\right)=\kappa e^{-\eta \cdot \left(t-t_{0}\right)} \end{array}$$

in which, η is the descend factor and related to time. κ is the initial strength*. t*_0_ is the begin moment. A little time will be taken for large convergence strength and long for a small value. So, the convergence process is mainly determined by the state of *k*_*e*_(t) > = 1, then the effective time *T*_*effective*_ of convergence can be solved by

(26) $$\begin{array}{r} e^{-\eta \cdot \left(t-t_{0}\right)}\geq 1/\kappa \end{array}$$

(27) $$\begin{array}{r} T_{effective}=\left(t-t_{0}\right)\geq \ln\;\left(\kappa \right)/\eta . \end{array}$$

**Figure 6:**
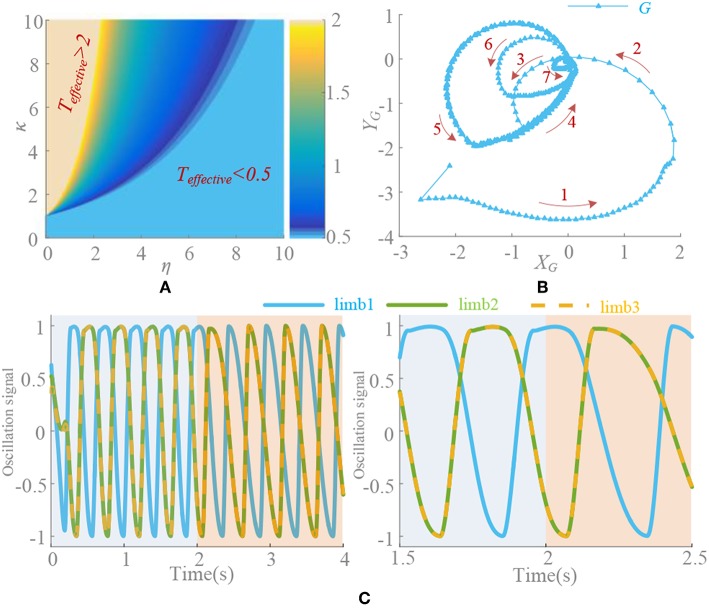
**(A)** Effective time surface of *k*_*e*_(t). *T*_*effective*_>2s and *T*_*effective*_ <0.5s is marked. *T* = 0.5s. **(B)** Convergence process. Red arrow is the direction of limit circle. **(C)** Deformation elimination. *k*(t) = 5 in 0–2 s and changes to *k*_*e*_(t) at 2 s (*t*_0_ = 2 s). ρ = 5/6, κ = η = 5, *t*_0_ = 2 s, ψ = [0, 0.5, 0.5, 0, 0, 0.5] and signal of limb 1, 2, and 3 are selected.

Therefore, in the steady state, when *k*(*t*) = 0, the distortion vanishes and the period recovers to normal. The problem of oscillation deformation caused by the asymmetry factor is solved. However, the adjustment capability of synchronization is not available at this moment. If the phase difference is inaccurate as required before *k*(*t*) = 0, it cannot be adjusted any more. Therefore, choosing the right parameters to ensure fast and stable convergence is important. It can be seen from the Figure 6 that κ and η are approximately linearly correlated. According to the finds above, when κ is too large, deformation must not be ignored. Then κ and η are better taking values within [1, 10]. In this paper, κ = η = 5 and the convergence time is 0.32 s. Finally, the σ-Hopf oscillators with synchronization used in the system is

(28) $$\begin{array}{r} {\overset{∙}{\upsilon }}_{i}=\text{f}\left(\upsilon_{i},\mu_{i},\sigma_{i}\left(\rho_{i},\lambda ,t\right)\right)-\kappa e^{-\eta \cdot \left(t-t_{0}\right)}.\\ \;\overset{n_{i}}{\underset{j\in {𝔑}_{i}}{\sum }}\left(\upsilon_{i}-\frac{\mu_{i}}{\mu_{j}}\text{R}\left(\Delta \Phi_{ij}\right)\upsilon_{j}\right)+\text{u}\left(t\right) \end{array}$$

in which, the positive scalar κ denotes the coupling gain and can be time-varying for different locomotion, η denotes the descend factor, and *t*_0_ is the start point.

## Neurobiologically Inspired Control and Results

The forward and inverse kinematics of parallel robots is a cumbersome task, and there are a lot of related researches (Merlet and Pmerlet, 2006; Li and Wen, 2011; Huang et al., 2013). So, the presented CPG signal processing and network construction is used for parallel bionic waists in this section. It can effectively avoid complex solving processes and achieve coordinated bionic behavior. But the formation and construction of coupling bionic behavior of such parallel structures is still unknown. The waist is mainly composed of lumbar bones and muscles. It helps the torso to achieve multi-directional bending and axial torsion and transmits (spinal) nerve signals and loads of legs (Hashemirad et al., 2009; Galis et al., 2014). The proposed parallel waist can achieve identical functions, which provide a structural basis for the control strategy. In this paper, four main movements: stretch, lateral shift, pitch and torsion (motion pattern A, B, C, and D) are discussed. In order to prove the effectiveness of the method, the range deviation, motion transformation characteristics, and position error of the waist will be analyzed from the amplitude, phase, and deformation of the oscillation signal, respectively.

### Amplitude and Frequency

**Figure 7:**
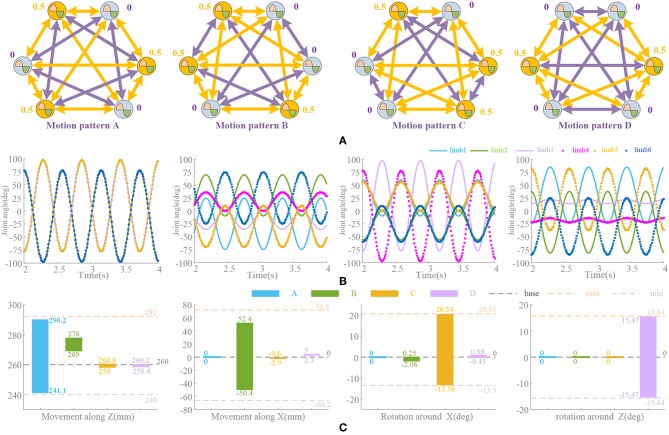
Different motion patterns in different directions. **(A)** Network configuration. Coupling factors are set according to Materials and Method. Yellow arrows link oscillators with 180° phase difference. Phase factors (ψ_*i*_ = Φ_*i*_/2π) is marked. Purple arrows mean no phase difference. **(B)** Amplitude and offset of oscillation signals. **(C)** Deviations between neurobiologically inspired control and kinematics solution [the forward and inverse kinematics formula and structure parameters are shown in Appendix (Supplementary Material)]. Movement along and around axial Y is not taken into consideration for the symmetry of structure and movement.

According to the bionic waist structure and coupling network established in Materials and Method, the phase relationship of the six oscillation signals is shown in Figure 7A. These signals will be directly used to drive the limbs of the platform. Due to the problem of multiple solutions in the inverse kinematics of parallel robots (Ahmet and Koksal, 2014), repeat movements occur in the full rotation range of the actuator. That is to say, the different configurations of actuator can result in the same position of the moving platform, so the appropriate range of joint rotation must be selected, which satisfies both the maximum movement space and the requirement of smooth locomotion. Compared to the series structure, it is not so simple to achieve independent movement in the parallel bionic waist, since the movement of a single actuator will cause multiple degrees of freedom of the moving platform. Learning from the forward and inverse kinematics of the parallel robot and the structural characteristics of the bionic waist (see Appendix in Supplementary Material), the amplitude and offsets of the oscillation signal are tuned. The control waveform shown in Figure 7B is a result of the amplitude and phase adjustment for the range and direction of the locomotion.

In order to verify the effectiveness of the motion synthesis, the limit range of movement under CPG based control is compared with that obtained by kinematics solution. The deviations of the main motions are shown in Figure 7C. Among them, the actuator range is referenced to the maximum motion position and distributed according to the oscillator waveform (approximate linear distribution). However, the actual parallel platform motion is decomposed into a rotation of 6 actuators with strong non-linear coupling property, which depends on the complexity of motion synthesis, so there are movements in other directions besides the main control directions, i.e., almost every motion pattern will cause movements along axial Z. If the pre-processing module or network is used for non-linear mapping and planning, this phenomenon can be improved and more complicated locomotion can be realized, but in this paper the limbs are directly driven by the oscillation signals at present.

With regard to frequency, most of the motions in quadruped mammals are in low frequencies, and even high-speed running will not carry out in too high frequency. The frequency transition problem has been discussed in detail (Nachstedt et al., 2017). Since the parallel waist frequency does not change too much and has no abrupt change, it will not cause waveform deformation and position deviation. Of course, in order to improve the adaptability, optimization and adaptive methods can be used for frequency adjustment, and there are many methods (Righetti et al., 2006; Nachstedt et al., 2017).

### Phase

**Figure 8:**
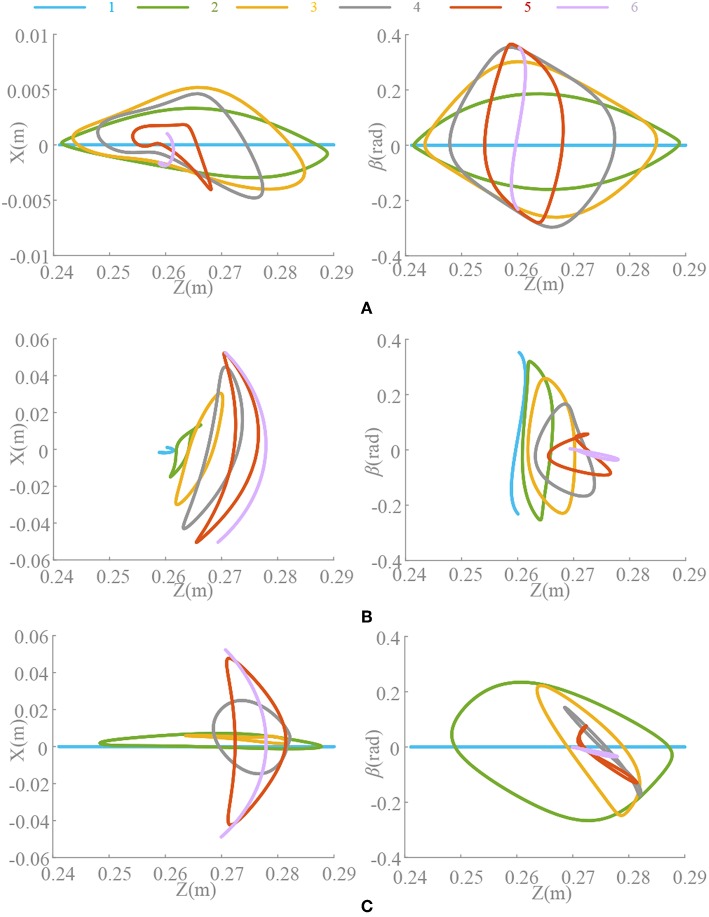
Transformation of different motion patterns. β is the pitch angle round Y. **(A)** Motion pattern A → motion pattern C. **(B)** Motion pattern C → motion pattern B. **(C)** Motion pattern A → motion pattern B. All transformation is conducted in 5 steps. Both phase and amplitude changes linearly.

According to the foregoing illustration, the phase differences in the four typical motions have been determined by the structure and the motion state. In nature, such states in mammals are very easy to transform, i.e., the stretching (motion pattern A) of the torso is easy to transform into a lateral shifting motion (motion pattern B) or a pitching motion (motion pattern C). While the motion pattern D is relatively independent. Generally, it is necessary to return to the initial state before performing other locomotion. According to the symmetric distribution of the actuators (Δ_16_ = Δ_25_ = Δ_34_ = 180°), the motion patterns A, B, and C can be simplified to consider the phase difference of the single-sided actuator 1–2–3. According to Figure 8, three changes are conducted: A → C, C → B, and A → B. A → C and C → B can be supposed as similar locomotion, since only one group of oscillators needs to change phase, indicating that most of the actuators have similar movements in the two motion patterns. Therefore, the transformation should be very smooth. But A → B needs to change two sets of oscillators which cannot be a similar motion. If a transformation like A → B is unavoidable, it is best to change through similar motion, such as A → C → B through a bending motion or A → AB → B through a coupling transition. It is recommended to change state between adjacent motion patterns. Otherwise, it will cause large deviation and even malfunction. In the transformation, the oscillation amplitude should be adjusted at the same time, since it is related to both the changing process and result.

CPG-based locomotion control with phase difference adjustment enables fast transformation between motion patterns and formation of several coupling motions. Without a doubt, most of the mammalian movements are a synthesis of simple movements, i.e., the running process is the synthesis of motion pattern A and motion pattern C. In addition, it is worth mentioning that the motion pattern of the parallel waist is not only determined by the phase difference of the oscillator, but also by the amplitude.

### Duty Factor ρ

**Figure 9:**
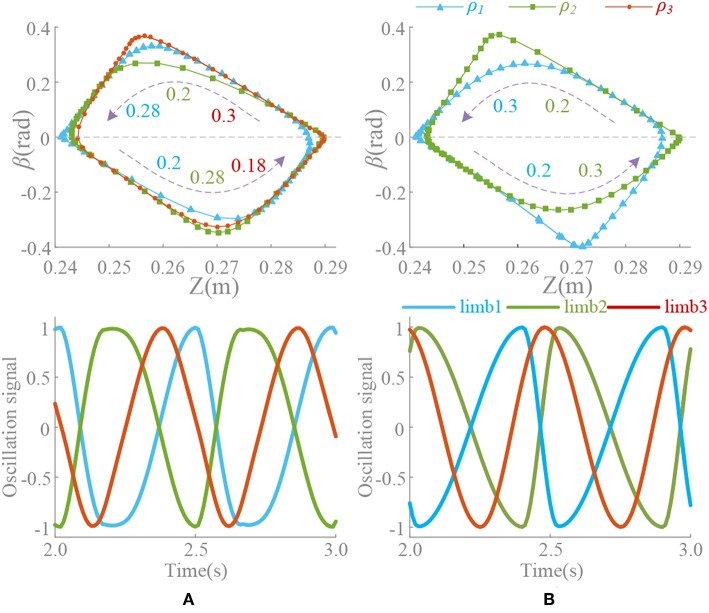
Position trajectory and waveform of coupling motion. *T* = 0.5 s, ψ = [0, 0.5, 0.2, 0.3, 0, 0.5], ρ_1_ = [3/4, 1/4, 0.55, 0.45, 3/4, 1/4], ρ_2_ = [1/4, 3/4, 0.45, 0.55, 1/4, 3/4], ρ_3_ = [3/4, 1/4, 3/4, 1/4, 3/4, 1/4]. Forward duration (below zero line) and back duration (above zero line) are influenced by ρ^*limb*^^3^, which is corresponding to limb 3 and Δ_13_. β is the pitch angle round Y. **(A)** Constant coupling strength, *k*(*t*) = 5. **(B)** Proposed coupling strength *k*(*t*) = *k*_*e*_(*t*).

The significance of introducing σ is to make an asymmetrical duty cycle in oscillation, so that the locomotion is more controllable and coordinated, such as the leg swinging of walking robot (Xiong et al., 2017), the wings flapping of flying robot (Briod et al., 2014), and the fin swaying of underwater robots (Wang et al., 2019). But the asymmetry oscillation always brings about deformation of the waveform as well as the period changes, which has been discussed in Materials and Method and Analysis of Synchronization. And we noticed that selection of ρ is also influenced by the phase difference, i.e., in the Z-direction motion, the difference between the oscillator 1 and the oscillator 2 is 180°. Therefore, in order to maintain the consistent action, if the ρ of the oscillator 1 is 3/4, the ρ of the oscillator 2 should be 1/4. Thus, the locomotion coordination can be ensured. So, if the motion transformation is performed, the value of ρ must also be changed correspondingly. A fixed ρ value may not satisfy the phase difference requirement. The coupling motion of motion pattern A and C is analyzed in Figure 9. In the transformation from motion pattern A to C (Δ_13_ = 0 → Δ_13_ = 0.5), ρ should do the same change.

During the above motion, the existence $G'\left\{\text{G}\left({\bar{\upsilon }}_{0}\right),k\left(t\right)\right\}$ in the (21) leads an unexpected change on period of oscillation signal in the steady state. Unsuitable ρ will make it worse. ρ^*limb*^^3^ keeping unchange (ρ^*limb*^^3^ = 3/4) through the transformation (Δ_13_ = 0.2) leads an unpredictable forward-back duration ratio shown in Figure 9A. This effect cannot be eliminated if *k* is non-zero, which reduces the control ability of ρ to the waveform. Moreover, the effect or the change is actually difficult to detect or predict. The method of this paper can control the waveforms of each oscillator as well as the ρ and phase difference, so that some special motion points even can control effectively shown in Figure 9B. The value of ρ should be adjusted timely according to the phase difference to make the transformation more coordinated and less impact. Furthermore, precise control and smooth motion are to be further improved by optimizing control and learning, evolution and other methods.

## Simulation

**Figure 10:**
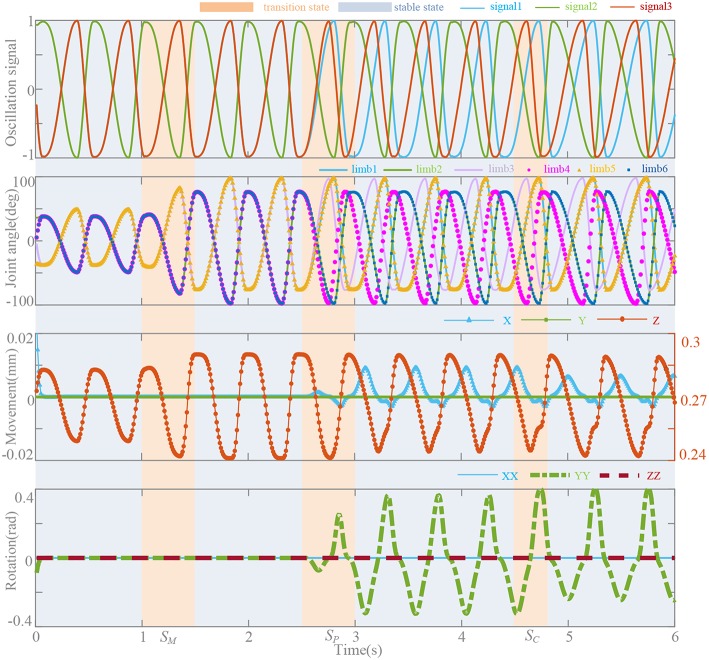
Oscillation signals, joint angles, and locomotion trajectories. *T* = 0.5 s, *k*(*t*) = 5, ψ = [0, 0.5, 0, 0.5, 0, 0.5], and ρ = [1/4, 3/4, 1/4, 3/4, 1/4, 3/4]. Magnitude transition (*S*_*M*_): movement Z (0.25, 0.28 mm) → (0.241, 0.292 mm). Phase transition (*S*_*P*_): ψ → [0, 0.5, 0.2, 0.7, 0, 0.5]. Coupling strength transition (*S*_*C*_), *k*(*t*) = 5 → *k*(*t*) = *k*_*e*_(*t*).

To investigate the effects of neurobiologically inspired control method on bionic parallel waist, we conducted co-simulation by MATLAB/ADAMS. The parameters of the parallel platform are shown in the Appendix (Supplementary Material). The control frame is presented in Platform and System and shown in Figure 1C. With the bionic control method, control signals for the motor driver are generated by the oscillators and synchronous network. Specifically, we set 3 transition states for magnitude (*S*_*M*_), phase (*S*_*P*_), and coupling strength (*S*_*C*_) to evaluate the performance of the method. Oscillation signals, joint angles, and moving platform locomotion trajectories including movements and rotations are shown in Figure 10. Before 2.5 s, the platform performs in motion pattern A, so there are only movements along Z axial. The magnitudes are regulated by linear transformation in 0.5 s and they make the moving distance change from 0.25–0.28 mm to 0.241–0.292 mm. To perform a bending movement (rotation around Y axial), **ψ** is gradually set from [0, 0.5, 0, 0.5, 0, 0.5] to [0, 0.5, 0.2, 0.7, 0, 0.5] in 0.5 s from the moment at 2.5 s to regulate the phases of limb 3 and 4. This leads the moving platform to rotate around Y axial and slight movements along X axial. In the coupling strength transition at 4.5 s, coupling strength *k*(*t*) = 5 turns to *k*(*t*) = *k*_*e*_(*t*) and signals become stable in 0.32 s according to the effective time surface of *k*_*e*_(t). The duty factor is set constant as ρ = [1/4, 3/4, 1/4, 3/4, 1/4, 3/4] and the distortion begin to diminish at 3.5 s when *k*(*t*) = *k*_*e*_(*t*). Influence on the movements and rotations of this factor also can be seen in Figure 9.

## Conclusion

A synchronous bionic control strategy based on σ-Hopf for a bionic parallel waist is proposed. To identify and evaluate waveform distortion of asymmetry σ-Hopf oscillation in synchronization, the transition state is analyzed. The variable coupling strength is raised to eliminate distortion and ensure effective and stable synchronization simultaneously. On this basis, the bionic control network for the parallel waist is constructed to realize the typical behavior. The effects of amplitude, frequency, phase, and duty factor on the behavioral deviation, motion pattern transformation, and forward-back duration adjustment are discussed. The main contributions of this paper are:

1. σ-Hopf oscillator has a symmetrical circular limit cycle and the waveform is regular and stable. Different from other oscillators, its frequency, amplitude, and duty ratio can be adjusted independently, which contribute to the control of different moving speeds, motion range, and forward-back duration. These advantages can enhance behavioral diversity and agility of bionic robot, which is of great significance for bionic control.
2. The waveform characteristics of σ-Hopf oscillator makes the synchronization process more complicated than symmetric oscillator. Our analysis and methods show a way to eliminate the deformation and ensure the fast convergence. Thus, without changing the characteristics of σ-Hopf oscillator, synchronization process is achieved and can be used for the coordinated behavior and coupled locomotion of the multi-joint bionic robot.
3. The σ-Hopf oscillator based neurobiologically inspired control is put forward to enhance the controllability for the bionic waist, which has really strong coupling characteristics. Not only the initial and final state of motion, but also the intermediate state and instantaneous state can be controlled precisely. This will benefit the transition and transformation of the locomotion and makes the coupling motion more flexible.

In this paper we provide a waveform-controllable oscillator and an undistorted synchronization method for the bionic robot, i.e., legged robot, flying robot, and swimming robot, etc. In the future, we will build the entire motion control network of the quadruped robot with bionic waist for coordinated motion. As the network architecture becomes more complex, the proposed method will make more sense for the controllable locomotion and synchronization process.

## Data Availability

All datasets generated for this study are included in the manuscript and/or the Supplementary Files.

## Author Contributions

YZ designed the method. YZ, SZ, DG, and QL designed and performed the simulations. YZ and SZ analyzed the data and wrote the paper.

### Conflict of Interest Statement

The authors declare that the research was conducted in the absence of any commercial or financial relationships that could be construed as a potential conflict of interest.

## Footnotes

^1^https://www.bostondynamics.com/robots.
