# Supplementary material for: Synchronization of Non-linear Oscillators for Neurobiologically Inspired Control on a Bionic Parallel Waist of Legged Robot
Source: Front Neurorobot. 2019 Aug 2;13:59. doi: 10.3389/fnbot.2019.00059 (PMC6687854; doi:10.3389/fnbot.2019.00059)
Supplement: Supplementary file 1 [file Data_Sheet_1.docx]

*Appendix of Synchronization of Nonlinear Oscillators for Neurobiologically Inspired Control on a Bionic Parallel Waist of Legged Robot*

In this Appendix we will show the inverse and forward kinematics of 6-RSS parallel platform.

## 1. Inverse Kinematics

Geometric configuration of the parallel platform is shown in the Fig. 1, 6 motors are distributed in three groups (1-6, 2-3, 4-5) .The coordinate system {*B*} and {*P*} are attached on the stationary platform and moving platform, respectively. $S_{i}^{P}$ and $S_{i}^{B}$ denote the center point of spherical joint 1 and 2, $R_{i}^{B}$is center point of the revolute joint of motor, *i* denotes the *i*th limb. Other structure parameters are shown in TABLE.

Fig. 1. Geometric structure of parallel platform.

TABLE Structure parameters

| Symbol | Structural parameters | Value |
| --- | --- | --- |
| *γ* | motor tilt angle | π/6 *rad* |
| *R_0_* | radius of *S^P^* circle | 0.098 *m* |
| *R* | radius of *R^B^* circle | 0.140 *m* |
| *L* | group distance of *R^B^* | 0.070 *m* |
| *L*_1_ | group distance of *S^P^* | 0.024 *m* |
| *r* | input rod length | 0.023 *m* |
| *L_2_* | coupler link length | 0.250 *m* |
| *L_3_* | height of *S^P^* | 0.013 *m* |
| *h* | height of *R^B^* | 0.032 *m* |

The vector $O^{B}R_{i}^{B}$ and $O^{P}S_{i}^{P}$ can be obtained directly from the structure parameters.

$O^{B}R_{i}^{B}=[R\cos\omega_{i},R\sin(\omega_{i}),h]$, (1)

$O^{P}S_{i}^{P}=[R_{0}\cos\sigma_{i},R\sin\sigma_{i},-L_{3}]$, (2)

$\omega_{i}=\{-\alpha,-\frac{2\pi}{3}+\alpha,-\frac{2\pi}{3}-\alpha,-\frac{4\pi}{3}+\alpha,-\frac{4\pi}{3}-\alpha,\alpha\}$, (3)

$\sigma_{i}=\{-\frac{\pi}{3}+\beta,-\frac{\pi}{3}+\beta,-\pi+\beta,-\pi-\beta,\frac{\pi}{3}+\beta,\frac{\pi}{3}-\beta\}$, (4)

$\alpha\text{=}\arcsin\frac{L}{2R}$,$\beta\text{=}\arcsin\frac{L_{1}}{2R_{0}}$. (5)

If the position and orientation [*X*, *Y*, *Z*, *ϕ*, *θ*, *ψ*] of moving platform respect to {*B*} is known, the vector $O^{B}S_{i}^{P}$ from *O_B_* to *S^P^* with respect to {*B*} is

$O^{B}S_{i}^{P}={}_{P}^{B}T\times O^{P}S_{i}^{P}+C(i=1,2\ldots6)$ (6)

(7)

where, sin and cos are written as *s* and *c*. *C* is the position vector of *O_P_* in the {*B*}. ${}_{P}^{B}T$ is the rotation matrix between the {*P*} and {*B*}. Direction vector of the axis of the motor output shaft with respect to {*B*}

$U_{i}=[\frac{h}{\tan\gamma}\cos\varsigma,\frac{h}{\tan\gamma}\sin\varsigma,h]$ (*i*=1, 2…6), (8)

where $\varsigma=\{0,-\frac{2\pi}{3},-\frac{2\pi}{3},-\frac{4\pi}{3},-\frac{4\pi}{3},0\}$, the orientation of each group of motor corresponds to *α*. Let us define three vectors

$X_{i}^{B}=\left\{\begin{matrix} \frac{U_{i}\times Z^{B}}{|U_{i}||Z^{B}|},i=1,3,5 \\ \frac{Z^{B}\times U_{i}}{|U_{i}||Z^{B}|} ,i=2,4,6 \end{matrix}\right.$,$Y_{i}^{B}=\frac{U_{i}\times X_{i}^{B}}{|U_{i}||X_{i}^{B}|}$,$Z^{B}=[0,0,1]$. (9)

Then, according to the vectors on each limb, we find

$R^{B}S_{i}^{B}=r(X_{i}^{B}\cos\theta_{i}+Y_{i}^{B}\sin\theta_{i})$ (10)

$R^{B}S_{i}^{p}=O^{B}S_{i}^{P}-O^{B}R_{i}^{B}$ (11)

$S^{B}S_{i}^{P}=R^{B}S_{i}^{P}-R^{B}S_{i}^{B}$. (12)

The distance between $S_{i}^{P}$ and $S_{i}^{B}$ is

$\left|S_{i}^{B}S_{i}^{P}\right|^{2}=L_{2}^{2}=r^{2}-2R_{i}^{B}S_{i}^{P}\cdot R_{i}^{B}S_{i}^{B}+\left|R_{i}^{B}S_{i}^{P}\right|^{2}$. (13)

We define $\Lambda_{i}=L_{2}^{2}-r^{2}-\left|R_{i}^{B}S_{i}^{P}\right|^{2}$ to synthesize the above equations. Then,

$\Lambda_{i}+2R_{i}^{B}S_{i}^{P}\cdot r(X_{i}^{B}\cos\theta_{i}+Y_{i}^{B}\sin\theta_{i})=0$. (14)

If we define$\chi=\tan(\theta_{i}/2)$, it can be rewritten as

$\chi^{2}(\Lambda_{i}-2rX_{i}^{B}\cdot S_{i}^{B}S_{i}^{P})+\chi(4rY_{i}^{B}\cdot S_{i}^{B}S_{i}^{P})+2rX_{i}^{B}\cdot S_{i}^{B}S_{i}^{P}+\Lambda_{i}=0$. (15)

Thus, the equation can be solved and *θ_i_*=2arctan*χ*, there are two solutions here.

## Forward Kinematics

To show the difference in forward kinematics, is used for all the variables. In the same way, the vectors are

$\hat{R}_{i}^{B}\hat{S}_{i}^{B}=\hat{r}(\hat{X}_{i}^{B}\cos\hat{\theta}_{i}+\hat{Y}_{i}^{B}\sin\hat{\theta}_{i})$, (16)

$\hat{O}^{B}\hat{S}_{i}^{B}=\hat{O}^{B}\hat{R}_{i}^{B}+\hat{R}_{i}^{B}\hat{S}_{i}^{B}$, (17)

$\hat{S}_{i}^{B}\hat{S}_{i}^{P}=\hat{O}^{B}\hat{S}_{i}^{P}-\hat{O}^{B}\hat{S}_{i}^{B}$. (18)

The length *L_2_* of the six coupler links is consistent, so

$L_{2}^{2}=\left|\hat{O}^{B}\hat{S}_{i}^{P}-\hat{O}^{B}\hat{S}_{i}^{B}\right|^{2}$. (19)

Since the angle $\hat{\theta}_{i}$ of the revolute joint is known, the position and orientation of the platform can be solved by equations

$\text{|}{}_{P}^{B}\hat{T}\times\hat{O}^{P}\hat{S}_{i}^{P}+\hat{C}-\hat{O}^{B}\hat{R}_{i}^{B}+r\cos\hat{\theta}_{i}\hat{X}_{i}^{B}+r\sin\hat{\theta}_{i}\hat{Y}_{i}^{B}\text{|}^{2}-|L_{2}|^{2}=0,i=1,2,3,...,6$ (20)

Newton-Raphson method is used to s find out the solution of .
